# Opium Habit

**Published:** 1877-09

**Authors:** 


### OPIUM HABIT.

We occasionally receive letters of inquiry from physicians in various parts of the country in regard to opium antidotes. To these, and to all who are interested on this subject, we desire to express our opinion without reserve.

The habit of using opium constantly is certainly one of the most injurious to the subject, of which we have any knowledge, not excepting alcoholic drinks. The mind and body become a wreck from which they are never restored without very great difficulty. The cause of humanity is therefore most effectually served by any succesful plan of relieving these unfortunate sufferers.

Secret nostrums are being sent all over the country in large amount, and at enormous prices, for this purpose. The victims of this habit and of the vender's schemes, eagerly seize upon any promised relief, particularly when shrouded in mystery.

Physicians find great difficulty in controlling such patients in the use of proper means, and sometimes feel constrained to encourage secrecy in order to have them continue the remedy.

Collins and others who have found immense profit in their secret "opium antidote," doubtless use the same article as the principle ingredient in their nostrums. Opium no doubt forms the basis of all, and the cure is effected by *gradual reduction.* Proof of this is found in the fact that gradual reduction carried out as faithfully as the nostrum is taken, proves equally successful; and indeed more speedy in the cure, on account of the slow decrease in quantity which some of the nostrum dealers require. Other neurotics have been detected, and are doubtless added to these nostrums. Strychnine probably enters into all, and quinine perhaps in some. These with opium, some mineral acid, and aniline for coloring, make up the composition of the average "painless opium antidote."

If a practicing physician tells an opium eater that he must gradually decrease the quantity and lengthen the interval between the doses, in order to break up the habit and restore him to usefulness, health and pleasure, he often fails to carry out the plan arranged for him. The advertisement of the charlatan meets his eye, the charm of secrecy lays hold on him, and he pays enormous prices for concealed morphine and strychnine.

What is the duty of physicians, under these circumstances? This is the great question to be answered. Should we encourage the purchase of these humbug preparations, or supinely allow our patient to be thus deceived without an effort to convince him and the public of the deception?

If exposure of this fraud would redound to the pecuniary benefit of the physician alone, of course the public would not be especially interested in becoming enlightened on the subject, but since the greater benefits are to be secured to the unfortunate subjects of the habit, we have no hesitation, as a journalist, in proclaiming the truth on this subject. If our neighbors who are the subjects of this vice can greatly profit by paying us a moderate fee to conduct them out of the difficulty, it is a duty we owe them as well as ourselves, to induce them to take this course.

Exactly what amount of benefit is derived from the addition of strychnine to the preparation of opium used in the process of destroying the habit, is not generally known, but that advantage is obtained from it, and perhaps quinine also, seems now to be very satisfactorily established by those who have tested them in this way.

The plan of some, is to increase the strychnine as the opium is decreased, until an amount of the former as large as the patient can bear without unpleasnat effects, has been arrived at.

We have no doubt that the most inveterate opium eater may be cured of the habit in a few months, by the use of strychnine in this way, and the decrease in quantity and lengthening of interval between the doses of opium.

We have freqently published analyses of nostrum opium antidotes and the reports of cures by the plan above suggested, and shall continue to do so as they are presented. Physicians generally agree on this subject, and we hope the public will soon be informed sufficiently to receive the benefits of advancement in medicine.

